# Depressive Symptoms and Physical Activity Mediate the Adverse Effect of Pain on Functional Dependence in Patients With Arthritis: Evidence From the Canadian Longitudinal Study on Aging

**DOI:** 10.1093/ptj/pzaf120

**Published:** 2025-10-03

**Authors:** Miriam Goubran, Zachary M van Allen, Martin Bilodeau, Matthieu P Boisgontier

**Affiliations:** Faculty of Health Sciences, University of Ottawa, Ottawa, Ontario, Canada; Bruyère Health Research Institute, Ottawa, Ontario, Canada; Faculty of Health Sciences, University of Ottawa, Ottawa, Ontario, Canada; Perley Health Centre of Excellence in Frailty-Informed Care, Ottawa, Ontario, Canada; Faculty of Health Sciences, University of Ottawa, Ottawa, Ontario, Canada; Bruyère Health Research Institute, Ottawa, Ontario, Canada; Faculty of Health Sciences, University of Ottawa, Ottawa, Ontario, Canada; Bruyère Health Research Institute, Ottawa, Ontario, Canada; Perley Health Centre of Excellence in Frailty-Informed Care, Ottawa, Ontario, Canada; Institut du Savoir Montfort, Hôpital Montfort, Ottawa, Ontario, Canada

**Keywords:** Aged Adults, Cohort Studies, Depression, Exercise, Mental Health, Rheumatology

## Abstract

**Importance:**

Arthritis is a chronic condition affecting hundreds of millions of people worldwide, often leading to pain and functional limitations.

**Objective:**

This study aimed to investigate the direct and indirect effects of pain on functional dependence in individuals with arthritis. Depressive symptoms and physical activity were examined as potential mediators of this relationship.

**Design:**

This study was a longitudinal cohort study.

**Setting:**

The study setting included community-dwelling adults participating in the Canadian Longitudinal Study on Aging.

**Participants:**

This study sample consisted of 6972 participants with arthritis, including 4930 with osteoarthritis and 694 with rheumatoid arthritis.

**Exposure:**

The exposure was the usual presence of pain or discomfort at baseline, with depressive symptoms (CESD-10) and physical activity (PASE) tested as mediators.

**Main Outcome and Measure:**

The main outcome was functional dependence in basic activities of daily living (ADL) and instrumental activities of daily living (IADL) at follow-up, measured with a modified version of the Older Americans’ Resources and Services Multidimensional Functional Assessment Questionnaire (OARS).

**Results:**

Baseline pain was positively associated with depressive symptoms (b = 0.356 [95% CI = 0.310 to 0.402]) and negatively associated with physical activity (b = −0.083 [95% CI = −0.125 to −0.042]). Functional dependence at follow-up was significantly predicted by baseline pain (log OR = 0.607 [95% CI = 0.261 to 0.952]), depressive symptoms (log OR = 0.358 [95% CI = 0.184 to 0.533]), and physical activity (log OR = −0.598 [95% CI = −0.818 to −0.378]). Mediation analysis showed that 23.3% of the total effect of pain on functional dependence was accounted for by the indirect effect through depressive symptoms (16.2%), physical activity (6.3%), and their serial combination (0.8%).

**Conclusions:**

The presence of pain at baseline was associated with higher odds of functional dependence in basic and instrumental activities of daily living after a mean follow-up period of 6.3 years, with depressive symptoms and lower physical activity acting as mediators.

**Relevance:**

The findings highlight the need for arthritis care to extend beyond pain management by incorporating strategies that address depressive symptoms and promote physical activity to preserve functional independence.

## INTRODUCTION

The global prevalence of arthritis, particularly osteoarthritis, has risen significantly in recent decades, with cases increasing by 137% from 256 million in 1990 to 607 million in 2021.^1^ If trends continue, projections indicate that 1 billion people will have osteoarthritis by 2050.^2^ As a result, the burden of arthritis on society will continue to grow, since pain, a primary symptom, has been shown to predict functional decline.^3–6^

**Figure 1:**
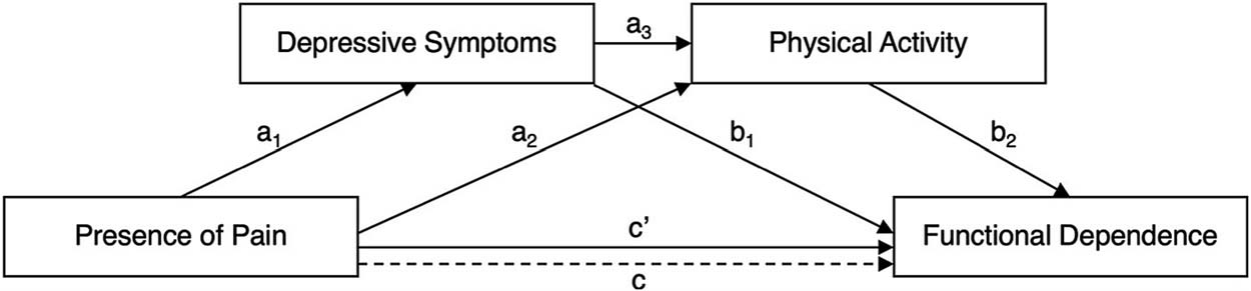
Path diagram of the serial mediation model testing the indirect effect of baseline pain on follow-up functional dependence via depressive symptoms and physical activity. a paths = effects of the independent variables on the mediators; a_1_ × b_1_ = indirect effect through depressive symptoms; a₂ × b₂ = indirect effect through physical activity; a_1_ × a_3_ × b₂ = indirect effect through depressive symptoms and physical activity; b paths = effects of the mediators on the outcome variable, controlling for the independent variable; c = total effect of pain when mediators are not adjusted for; c’ = direct effect of pain after adjusting for both mediators. all paths are adjusted for age, sex, and baseline functional dependence.

The current study builds on 2 theoretical frameworks explaining the relationship between pain and functional dependence. The fear-avoidance model describes how individuals who catastrophize pain may develop pain-related fear, leading to avoidance behaviors, physical disuse, depression, and disability.^7^ Similarly, the avoidance model, specifically applied to osteoarthritis, describes how pain and psychological distress can initiate a cascade starting with the avoidance of activities and leading to muscle weakness and activity limitations.^8^ More recently, meta-analytic evidence has further supported the association between fear of movement and reduced physical activity across clinical populations, including rheumatologic conditions such as arthritis.^9^ These models and results emphasize the role of psychological and behavioral variables in the pathway from pain to functional dependence. Informed by these frameworks, our study tested depressive symptoms and physical activity as mediators of the effect of pain on functional dependence in individuals with arthritis, using a longitudinal dataset (Figure 1). This empirical approach aimed to clarify the extent to which these mediators operate in parallel to influence functional dependence.

Understanding the relationship between pain and functional dependence has important implications, as loss of independence significantly affects quality of life and health care costs.^10^^,^^11^ While pain directly contributes to functional decline,^3–6^ this effect may be mediated by factors related to mental and physical health. Two relevant candidate mediators of the relationship between pain and functional independence are depressive symptoms and physical activity, as both are influenced by pain^12–16^ and predict functional dependence.^6^^,^^17^^,^^18^ Understanding the complex relationships between pain, depressive symptoms, and physical activity, and how they collectively influence functional dependence in individuals with arthritis may inform targeted interventions in this population.

Depression is one of the most common comorbidities in arthritis, with recent meta-analyses suggesting that it affects approximately one-third of this population.^19^^,^^20^ The relationship between pain and depressive symptoms has been consistently reported in the literature^21^ and may be bidirectional.^22^ Longitudinal studies investigating osteoarthritis have shown that greater pain symptoms prospectively predict the incidence of depression,^23–25^ while other studies have shown that depressive symptomology may exacerbate the experience of pain.^26^^,^^27^ However, in the context of arthritis, the mechanistic explanations supporting the effect of pain on depression are stronger than those supporting the reverse, mainly because pain is a primary and more direct symptom resulting from joint inflammation, whereas depression is a comorbidity that develops secondary to the chronic pain.

The mediation of the effect of pain on functional dependence by depressive symptoms is well documented in the pain literature,^28–35^ particularly in studies on back pain^28–31^ and traumatic injury.^32–34^ However, to the best of our knowledge, this mediation has not been examined in people with arthritis. This is a notable gap, given that arthritis is one of the leading causes of chronic pain and disability. Understanding the mechanisms underlying the relationship between pain and functional dependence in this population may inform interventions aimed at improving functional outcomes.

In contrast to the proposed model (Figure 1), studies in arthritis populations have primarily tested a different mediation model in which functional dependence is the mediator and depressive symptoms are the outcome,^36^^,^^37^ a model more relevant to mental health professionals. However, from a rehabilitation perspective, the focus is more on understanding the mechanisms that may improve functional outcomes. Moreover, conceptualizing depressive symptoms as a mediator aligns with the biopsychosocial approach to pain in rehabilitation,^38^ which emphasizes psychological contributions to physical functioning. The mechanistic basis for this mediation model is well supported, as depressive symptoms can reduce motivation,^39^ increase fatigue,^36^^,^^40^^,^^41^ and promote avoidance behaviors,^42^ all of which can affect physical function.^43^

Another potential mediator of the effect of pain on functional dependence is physical activity, as pain has been shown to predict physical activity levels,^44^ which in turn have been shown to improve physical functioning.^45^ While this indirect effect through physical activity has recently been examined in individuals with back pain,^46^^,^^47^ it has not been examined in individuals with arthritis. As physical activity has been shown to be an effective intervention for improving pain and function,^48^ refining our understanding of this relationship, accounting for the influence of depressive symptoms, could further inform rehabilitation programs for patients with arthritis.

The aim of this study was to examine the relationship between pain and functional dependence in people with arthritis using the Canadian Longitudinal Study on Aging dataset. Specifically, we tested whether depressive symptoms and physical activity mediate the effect of baseline pain on functional dependence at follow-up (Figure 1). We hypothesized that the presence of pain would have a direct effect on functional dependence. In addition, we hypothesized an indirect effect, whereby the presence of pain would be associated with higher depressive symptoms and lower physical activity levels, both of which would, in turn, predict higher odds of functional dependence, over and above the direct effect of pain.

## METHODS

### Participants

Participants in the Canadian Longitudinal Study on Aging (CLSA) were recruited by the CLSA team through Canada’s provincial health registries, random-digit dialing, and the Canadian Community Health Survey on Healthy Aging.^49^^,^^50^ Exclusion criteria for CLSA enrollment included residents living in Canada’s 3 territories and First Nations reserves, full-time members of the Canadian Armed Forces, individuals living with cognitive impairments, and individuals living in institutions, including 24-hour nursing homes.^49^ For the present study, we analyzed CLSA participants who had baseline measures of pain, depressive symptoms, physical activity, age, and sex, as well as measures of functional limitations at both baseline and follow-up. The study was approved by the University of Ottawa Research Ethics Board (H-10-23-9760).

Data of the baseline assessment was collected between 2010 and 2015 using 2 approaches: data collection from a tracking cohort of participants via 60-minute computer-assisted phone interviews and data collection from a comprehensive cohort via 90-minute in-person interviews in addition to a data-collection site visit. Additionally, a maintaining contact questionnaire was administered by phone to the comprehensive and tracking cohorts. The tracking cohort, comprehensive cohort, and maintaining contact questionnaire were used as baseline in our analyses. Between 2018 and 2021 another wave of data was collected during the follow-up assessment.

### Variables

#### Arthritis

At the baseline assessment, participants self-reported whether they had ever been diagnosed with rheumatoid arthritis, osteoarthritis, or any other type of arthritis. Five single-item questions were used: “Has a doctor ever told you that you have [osteoarthritis in the knee / osteoarthritis in the hip / osteoarthritis in 1 or both hands / rheumatoid arthritis / any other type of arthritis]?” Responses were yes or no. The dataset was initially filtered to include individuals with any type of arthritis for the main analyses. In addition, sensitivity analyses were conducted on 2 specific subsets: 1 comprising individuals with osteoarthritis and the other comprising those with rheumatoid arthritis.

#### Functional Dependence

Functional dependence in basic and instrumental activities of daily life (I/ADL) at baseline and follow-up was measured with a modified version of the Older Americans’ Resources and Services Multidimensional Functional Assessment Questionnaire (OARS).^51^^,^^52^ Twenty questions were asked to participants regarding their ability to complete 7 basic activities of daily life (ADL) (eg, can you eat without help, can you walk without help) in addition to 21 questions regarding 7 instrumental activities of daily living (IADL) (eg, can you use the telephone without help, can you prepare you own meals without help). The CLSA dataset includes a derived ordinal variable, the Basic and Instrumental Activities of Daily Living Classification that produces the following scores: 1 (no functional limitations), 2 (mild functional limitations), 3 (moderate functional limitations), 4 (severe functional limitations), and 5 (total functional limitations). This variable is derived from OARS responses using a method that assigns extra weight to the ability to prepare meals and aligns with variables used in Statistics Canada’s Canadian Community Health Survey.^53^ For our analyses, we computed a binary variable from this CLSA-derived classification at both baseline and follow-up. These variables classify participants with no or mild limitations (scores 1 and 2) as functionally independent, while those with moderate, severe, or total limitations (scores 3–5) were classified as functionally dependent. This binary variable was used in the statistical models.

#### Pain

The usual presence of pain or discomfort was assessed with the question “Are you usually free of pain or discomfort?” Responses were yes or no. We acknowledge that this item may reflect intermittent or persistent pain rather than constant presence or absence of pain, and that it captures both pain and discomfort. However, for readability, we often refer to this variable as “pain.”

#### Depressive Symptoms

The Center for Epidemiological Studies Short Depression Scale (CESD-10)^54^ was used to assess depressive symptoms at baseline. The CESD-10 contains 10 items measuring depressive feelings, restless sleep, hopefulness for the future, and loneliness (eg, “How often did you feel hopeful about the future?”; “How often did you feel that everything you did was an effort?”). Response options range from 3 [“All of the time (5-7 days)”] to 0 [“Rarely or never (less than 1 day)”]. A sum score ranging from 0 to 30 was used in the analyses. When 1 response was missing, CLSA imputed the value using the mean of the remaining 9 items to compute the sum score. Participants with more than 1 missing item were excluded from the analyses. Since the distribution for the CESD-10 was highly skewed, a log-transformed version of the variable was used in the analyses.

#### Physical Activity

Physical activity was measured at baseline with the Physical Activity Scale for the Elderly (PASE),^55^ which assesses the frequency of walking, light physical activity, moderate physical activity, strenuous physical activity, and exercise. Items asked participants to report on their activity levels over the previous 7 days on a 1 (never) to 4 (often, 5–7 days) scale and to indicate the time per day engaged in each of these activities on a 1 (less than 30 minutes) to 5 (4 hours or more) scale. A total physical activity score was derived from these items according to the PASE administration and scoring instruction manual,^56^ resulting in scores ranging from 0 to 485, with higher scores indicating higher levels of physical activity (see Suppl. Material S1 for scoring scheme).

### Statistical Analyses

All analyses were conducted in R version 4.4.1,^57^ and analysis scripts are publicly available.^58^ A serial mediation model, based on the process.R script (Model 6),^59^ was used to estimate total, direct, and indirect effects of baseline pain on functional dependence at follow-up. This regression-based approach^60^ was selected for its transparency and interpretability. To assess the robustness of the results, a sensitivity analysis based on structural equation modeling was conducted.

Functional dependence was assessed both at baseline (2011–2015) and at follow-up (2018–2021), while all the other variables were collected at baseline only. The independent variable was the usual presence of pain or discomfort (binary: 0 = usually free of pain, 1 = usually not free of pain), and the dependent variable was functional dependence at follow-up (binary: 0 = functionally independent, 1 = functionally dependent). The 2 mediating factors, physical activity and depressive symptoms, were measured as continuous variables. Untransformed values for the PASE (physical activity) and log-transformed values for CESD-10 (depressive symptoms) were standardized and included in statistical models in terms of standard deviations from their mean values in the cohort. All models were adjusted for age (standardized), sex (0 = female, 1 = male), and baseline functional dependence.

The total effect of pain on functional dependence (path c; Figure 1) was estimated using a logistic regression model in which pain was the predictor, adjusted for control variables (age, sex, baseline functional dependence). The direct effect (path c’) was estimated using a logistic regression model that was adjusted for the control variables and also included both mediating variables. Coefficients for path a_1_, a_2_, and a_3_ were estimated as β coefficients using linear regression. Coefficients for path b_1_ and b_2_ were estimated as log-odds coefficients (also known as logit coefficients or log odds ratios [log OR]) using logistic regression. The indirect effects were estimated using the product-of-coefficients approach. Because the outcome variable was binary, and in line with methodological recommendations,^59^^,^^61^^,^^62^ indirect effects were calculated on the log OR scale as the product of the β coefficient from the linear regression (path a) and the untransformed coefficient from the logistic regression (path b). Interpretation of log ORs follows the convention for logistic regression: positive values indicate increased odds, and negative values indicate decreased odds. Because individual path coefficients are estimated from linear and logistic regression models that use linear and nonlinear link functions, the estimates of direct and indirect effects may not sum to equal the total effect on the log odds ratio scale. Statistical inference for the indirect effects was conducted via empirical bootstrapping with 5000 resamples. This approach is recommended for multivariate non-normal data as it provides adequate control of Type I error rates and confidence interval coverage.^63^ Confidence intervals (95% CI) that did not include zero were interpreted as evidence of statistical significance.

The indirect effect of pain on functional dependence was estimated as the sum of 3 indirect pathways (Figure 1): pain → depressive symptoms → functional dependence (a_1_ × b_1_); pain → physical activity → functional dependence (a₂ × b₂); and pain → depressive symptoms → physical activity → functional dependence (serial pathway; a_1_ × a_3_ × b₂). Thus, the indirect effect of pain on functional dependence can be expressed as: Total indirect effect = (a₁ × b₁) + (a₂ × b₂) + (a₁ × a₃ × b₂).

Statistical significance was assessed using a 2-tailed α level of 0.05. The percentage of the total effect mediated by the mediators was calculated using the difference in log OR between the total effect (c) and the direct effect (c’), using the following formula:


\begin{equation*} Percentage\ Mediated=\frac{\log OR(c)-\log OR\left({c}^{{\prime}}\right)}{\log OR(c)}\times 100 .\end{equation*}

This calculation provides an alternative approach to quantifying mediation that does not rely on the individual components of the indirect effect described in the previous paragraph (ie, the products of specific pathways shown in Figure 1).

### Sensitivity Analyses

Four sensitivity analyses were conducted to assess the robustness of our results. The first sensitivity analysis used a different binary classification of functional dependence at follow-up, classifying participants with no functional limitations as functionally independent and those with mild, moderate, severe, or total functional limitations as functionally dependent. In the second sensitivity analysis, the functional dependence variable was redefined to exclude meal preparation, which may be less relevant for participants with lower limb arthritis. The third and fourth analyses were stratified by arthritis type, focusing respectively on participants with osteoarthritis and those with rheumatoid arthritis.

### Confirmatory Analysis

To validate the main mediation model, we conducted a supplementary analysis applying structural equation modeling using the lavaan package,^64^ returning to the original variable definitions and categories. This confirmatory analysis replicated the main model’s pathways and estimated indirect effects using an alternative estimation method (Suppl. Material S2).

### Role of the Funding Source

The funders had no role in the data collection, management, analysis and interpretation, writing of the report, or the decision to submit the report for publication.

## RESULTS

### Descriptive Results

A total of 6972 participants reported having arthritis, including 4930 with osteoarthritis, and 694 with rheumatoid arthritis. Among the 6972 participants with any type of arthritis (Table 1), baseline data showed that 58.7% were female participants, more than half of them reported experiencing pain or discomfort (52.8%), the mean (SD) physical activity score was 185.0 (75.8), and the mean (SD) depression score was 5.5 (4.6). The majority (90.5%) reported no functional limitations, while 8.8% had mild limitations, and fewer than 1% had moderate or severe limitations. Most participants were aged 55 to 64 years (37.5%), followed by those aged 45 to 54 years (24.7%) and 65 to 74 years (24.4%), with the smallest proportion in the 75 to 85 age group (13.5%). The distribution of demographic and clinical characteristics for participants with osteoarthritis and rheumatoid arthritis is presented in Table 1. The average time between baseline and follow-up measures was 6.3 (SD = 0.8) years, with a range of 4.0 to 9.6 years.

**Table 1:** Sample Characteristics at Baseline by Arthritis Type*^a^*

Variables	Any Arthritis (n = 6972)	Osteoarthritis (n = 4930)	Rheumatoid Arthritis (n = 694)
	Count (%) or Mean (SD)	Count (%) or Mean (SD)	Count (%) or Mean (SD)
Presence of pain			
No pain	3288 (47.2%)	2229 (45.2%)	270 (38.9%)
Pain	3684 (52.8%)	2701 (54.8%)	424 (61.1%)
Physical activity	185.0 (75.8)	180.2 (76.0)	190.9 (75.7)
Depressive symptoms	5.5 (4.6)	5.5 (4.7)	5.9 (4.8)
Sex			
Male	2882 (41.3%)	1943 (39.4%)	280 (40.3%)
Female	4090 (58.7%)	2987 (60.6%)	414 (59.7%)
Age group			
45–54 y	1720 (24.7%)	1002 (20.3%)	207 (29.8%)
55–64 y	2612 (37.5%)	1858 (37.7%)	236 (34.0%)
65–74 y	1698 (24.4%)	1332 (27.0%)	169 (24.4%)
75–85 y	942 (13.5%)	738 (15.0%)	82 (11.8%)
Functional limitations			
None	6310 (90.5%)	4417 (89.6%)	614 (88.5%)
Mild	611 (8.8%)	473 (9.6%)	71 (10.2%)
Moderate	43 (0.6%)	34 (0.7%)	5 (0.7%)
Severe	5 (0.1%)	4 (0.1%)	2 (0.3%)
Total	3 (0.05%)	2 (0.05%)	2 (0.3%)

^a^ This table reports raw, unstandardized, and untransformed values for physical activity that were obtained from the Physical Activity Scale for the Elderly (PASE) and values for depressive symptoms that were obtained from the Center for Epidemiological Studies Short Depression Scale (CESD-10). For use in regression models, raw PASE scores and log-transformed CESD-10 scores were standardized. The Functional Limitations variable reports counts of categories from the “Basic and Instrumental Activities of Daily Living Classification” derived in the Canadian Longitudinal Study on Aging dataset.

### Main Analyses

We used linear and logistic regression models to investigate the direct and indirect effects of baseline pain on functional dependence 6.3 (SD = 0.8) years later. Depressive symptoms and physical activity were examined as potential mediators. All analyses were adjusted for sex, age, and baseline functional dependence, with detailed results presented in Table 2. The main analyses were based on a sample 6972 participants with any type of arthritis.

**Table 2:** Regression Coefficients and Odds Ratios for the Mediation Paths in the Main Serial Mediation Model (n = 6972)*^a^*

Variables	**Path c** (Outcome = Functional Dependence)				**Path a**_**1**_ (Outcome = Depressive Symptoms)		
	Log OR	95% CI	*P*		b	95% CI	*P*
(Intercept)	−4.473	−4.822 to −4.150	<2 × 10^−16^		−0.137	−0.176 to −0.098	6.3 × 10^−12^
Age	0.839	0.684 to 0.997	<2 × 10^−16^		−0.072	−0.095 to −0.049	8.0 × 10^−10^
Sex	−0.291	−0.625 to 0.033	.082		−0.136	−0.183 to −0.089	1.1 × 10^−8^
Baseline dependence	3.439	2.810 to 4.068	<2 × 10^−16^		0.723	0.453 to 0.992	1.5 × 10^−7^
Presence of pain	0.791	0.458 to 1.138	4.8 × 10^−6^		0.356	0.310 to 0.402	<2 × 10^−16^
Variables	**Path a**_**2**_ **and a**_**3**_ **(Outcome = Physical Activity)**				**Path b**_**1**_ **, b**_**2**_ **, and c’** **(Outcome = Functional Dependence)**		
	**b**	**95% CI**	***P***		**Log OR**	**95% CI**	***P***
(Intercept)	−0.116	−0.151 to −0.082	5.2 × 10^−11^		−4.684	−5.042 to −4.326	<2 × 10^−16^
Age	−0.450	−0.470 to −0.430	<2 × 10^−16^		0.640	0.465 to 0.814	6.6 × 10^−13^
Sex	0.401	0.360 to 0.443	<2 × 10^−16^		−0.009	−0.348 to 0.329	.958
Baseline dependence	−0.744	−0.983 to −0.505	1.1 × 10^−9^		2.886	2.256 to 3.516	<2 × 10^−16^
Presence of pain	−0.083	−0.125 to −0.042	8.0 × 10^−5^		0.607	0.261 to 0.952	5.8 × 10^−4^
Depressive symptoms	−0.030	−0.050 to −0.009	5.3 × 10^−3^		0.358	0.184 to 0.533	5.7 × 10^−5^
Physical activity					−0.598	−0.818 to −0.378	9.4 × 10^−8^

^a^ b = regression coefficient (β); log OR = log(odds ratio).

#### Path c (Presence of Pain→Functional Dependence; Without Mediators)

**Figure 2:**
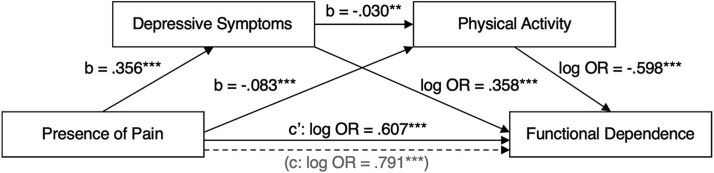
Path diagram displaying regression coefficients used to estimate direct and indirect effects of pain on functional dependence. b = regression coefficient β; c = total effect, c’ (dash arrow) = direct effect; log or = log(odds ratio). ^**^P < .01, ^***^P < .001.

Results showed a total effect (path c) of baseline pain on follow-up functional dependence (log OR = 0.791 [95% CI = 0.458 to 1.138]; *P* = 4.8 × 10^−6^) (Table 2, Figure 2). This model explained 15.0% of the variance in functional dependence (McFadden R^2^ = 0.150) (Table 2, Figure 2).

#### Path a1 (Presence of Pain→Depressive Symptoms)

Results showed that within each category of age, sex, and baseline functional dependence, participants with pain usually present at baseline had log-transformed scores on the CESD-10 depression scale that were on average 0.356 standard deviations higher (95% CI = 0.310 to 0.402; *P* < 2.0 × 10^−16^) than participants usually free of pain (Table 2, Figure 2). This model accounted for 4.7% of the variance in depressive symptoms (adjusted R^2^ = 0.047).

#### Path a2 (Presence of Pain→Physical Activity) and a3 (Depressive Symptoms→Physical Activity)

Participants with pain usually present at baseline engaged in less physical activity (path *a_2_)*, with PASE scores that were on average 0.083 standard deviations lower (95% CI = −0.125 to −0.042; *P* = 8.0 × 10^−5^) than participants usually free of pain. Similarly, participants with more depressive symptoms reported lower levels of physical activity (path a_3_). Specifically, participants who had log-transformed scores on the CESD-10 depression scale that were on average 1 standard deviation higher also had PASE physical activity scores that were on average 0.030 standard deviation lower (95% CI = −0.050 to −0.009; *P* = .005). Together, the variables in this model explained 25.3% of the variance in physical activity (adjusted R^2^ = 0.253) (Table 2, Figure 2).

#### Path b1 (Depressive Symptoms→Functional Dependence), b2 (Physical Activity→Functional Dependence), and c’ (Presence of Pain→Functional Dependence, with Both Mediators)

Accounting for age, sex, and baseline functional dependence, higher depressive symptom scores were associated with higher odds of functional dependence. Specifically, each standard deviation increase in log-transformed scores on the CESD-10 depression scale was associated with a 0.358-unit increase (95% CI = 0.184 to 0.533; P = 5.7 × 10^−5^) in log odds of being functionally dependent at follow-up, 4.0 to 9.6 years later (Table 2, Figure 2). Higher levels of physical activity at baseline were associated with lower odds of functional dependence at follow-up. Specifically, each standard deviation increase on the PASE was associated with a 0.598-unit decrease (95% CI = −0.818 to −0.378; P = 9.4 × 10^−8^) in the log odds of being functionally dependent (Table 2, Figure 2). Results showed a direct effect of baseline pain on functional dependence at follow-up (log OR = 0.607 [95% CI = 0.261 to 0.952]; *P* = 5.8 × 10^−4^) (Table 2, Figure 2), indicating that participants with pain at baseline had higher log-odds of being functionally dependent 4.0 to 9.6 years later compared to those without pain. This model accounted for 18.0% of the variance in functional dependence (McFadden R^2^ = 0.180).

### Indirect Effect

**Figure 3:**
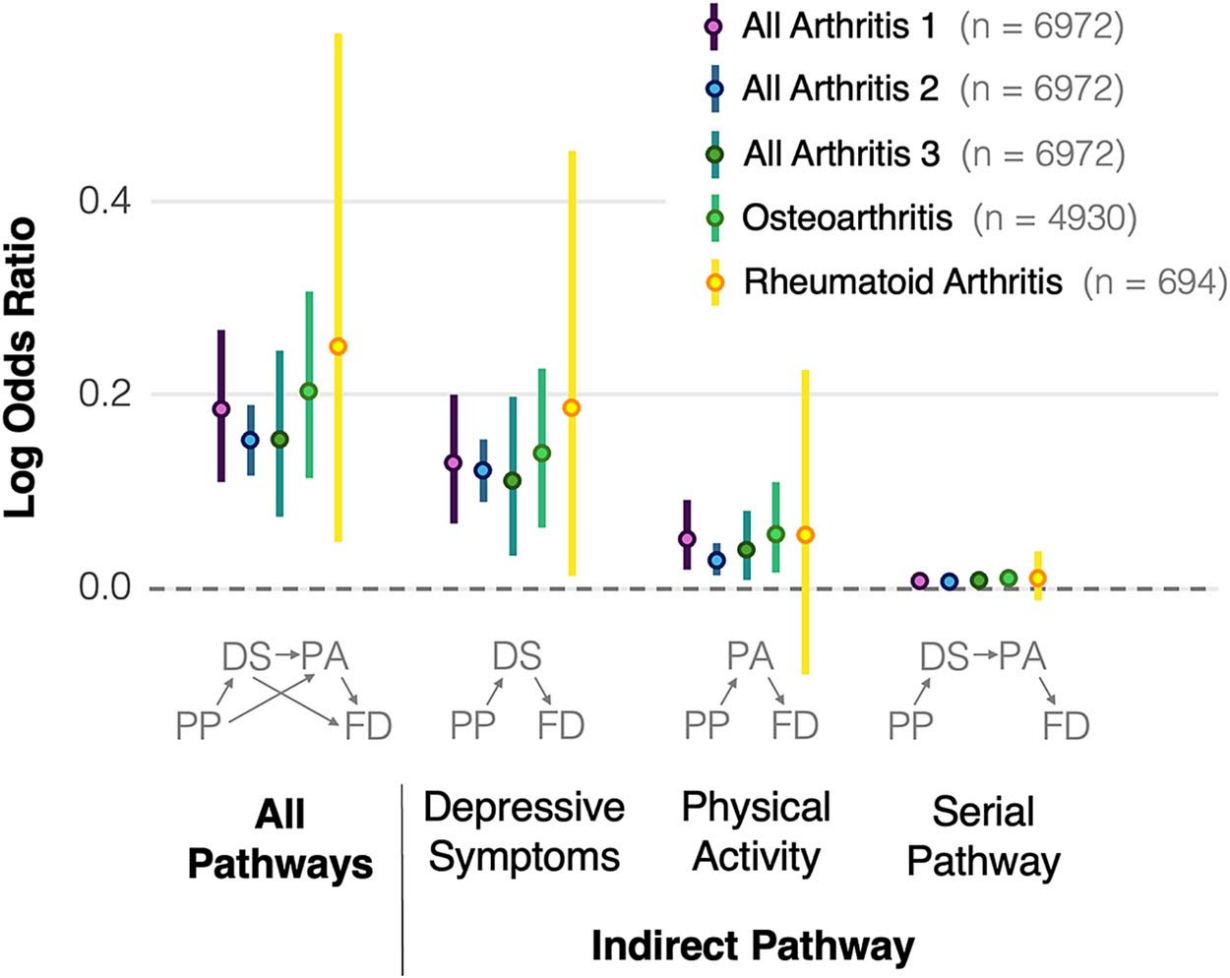
Indirect effect of baseline presence of pain on follow-up functional dependence. The indirect effect is partitioned into 3 indirect pathways through depressive symptoms (PP → DS → FD), physical activity (PP → PA → FD), and their serial combination (PP → DS → PA → FD). The horizontal dotted line at 0 indicates no effect, the dots represent estimated log odds ratios, and the vertical lines represent the 95% confidence intervals. Indirect pathways are presented for the main analysis, which included participants with any type of arthritis and classified them as functionally independent if they had no or mild functional limitations, and as functionally dependent if they had moderate, severe, or total functional limitations (All Arthritis 1). Four sensitivity analyses are also shown: participants with no functional limitations classified as functionally independent and those with mild, moderate, severe, or total functional limitations as functionally dependent (All Arthritis 2), measure functional dependence excluding meal preparation (All Arthritis 3), participants with osteoarthritis, and participants with rheumatoid arthritis. Abbreviations: DS = depressive symptoms; FD = functional dependence; PA = physical activity; PP = presence of pain.

Results based on the product of the path coefficients (a × b) showed an indirect effect of baseline pain on functional dependence at follow-up (log OR = 0.184 [95% CI = 0.111 to 0.265]), which was partitioned into 3 indirect pathways through depressive symptoms (log OR = 0.128 [95% CI = 0.064 to 0.200]), physical activity (log OR = 0.050 [95% CI = 0.019 to 0.090]), and their serial combination (log OR = 0.006 [95% CI = 0.001 to 0.013]) (Figure 3). The serial pathway with physical activity as the first mediator and depressive symptoms as the second mediator was also examined and yielded similar results (log OR = 0.0013 [95% CI = 0.0003 to 0.0028]). The exclusion of zero from all bootstrapped 95% CI indicated that each indirect effect was statistically significant.

The comparison of the total effect (path c) and the direct effect (path c’) showed that 23.3% of the total effect of baseline pain on functional dependence was mediated by the indirect effect through the mediators: 16.2% through depressive symptoms, 6.3% through physical activity, and 0.8% through the serial mediation of depressive symptoms and physical activity.

### Sensitivity Analyses

#### Different Classification of Functional Dependence

The sensitivity analysis that classified participants with mild functional limitations as functionally independent rather than functionally dependent, yielded results similar to those of the main analysis. Results showed an indirect effect of baseline pain on functional dependence at follow-up (log OR = 0.152 [95% CI = 0.117 to 0.189]) (Figure 3), which was partitioned into 3 indirect pathways: through depressive symptoms (log OR = 0.120 [95% CI = 0.089 to 0.154]), physical activity (log OR = 0.028 [95% CI = 0.013 to 0.046]), and their serial combination (log OR = 0.004 [95% CI = 0.001 to 0.007]).

#### Functional Dependence Excluding Meal Preparation

The sensitivity analysis based on a measure of functional dependence that excluded meal preparation yielded results similar to those of the main analysis. Results showed an indirect effect of baseline pain on functional dependence at follow-up (log OR = 0.154 [95% CI = 0.072 to 0.247]) (Figure 3), which was partitioned into 3 indirect pathways: through depressive symptoms (log OR = 0.112 [95% CI = 0.036 to 0.199]), physical activity (log OR = 0.038 [95% CI = 0.009 to 0.079]), and their serial combination (log OR = 0.005 [95% CI = 0.001 to 0.011]) (Figure 3).

### Osteoarthritis

Results of the sensitivity analysis focusing on participants with osteoarthritis (n = 4930) were similar to those of the main analysis. Results showed an indirect effect of baseline pain on functional dependence at follow-up (log OR = 0.203 [95% CI = 0.113 to 0.307]), which was partitioned into 3 indirect pathways: through depressive symptoms (log OR = 0.139 [95% CI = 0.062 to 0.227]), physical activity (log OR = 0.055 [95% CI = 0.016 to 0.109]), and their serial combination (log OR = 0.009 [95% CI = 0.002 to 0.019]) (Figure 3).

### Rheumatoid Arthritis

Results of the sensitivity analysis focusing on participants with rheumatoid arthritis (n = 694) suggested that the total mediation (log OR = 0.250 [95% CI = 0.049 to 0.574]) was primarily driven by the pathway through depressive symptoms (log OR = 0.185 [95% CI = 0.013 to 0.453]) as the pathway through physical activity (log OR = 0.055 [95% CI = −0.089 to 0.226]) and the serial mediation pathway through both depressive symptoms and physical activity (log OR = 0.010 [95% CI = −0.013 to 0.038]) had bootstrapped 95% CI that included zero (Figure 3), indicating that these indirect effects were not statistically significant.

### Confirmatory Analysis

A supplementary analysis using structural equation modeling (Suppl. Material S2) yielded results consistent with the main analysis in terms of the direction and significance of all paths. While estimates differed slightly in magnitude due to methodological differences, the overall pattern of direct and indirect effects was stable across approaches. These converging results further support the mediating roles of depressive symptoms and physical activity.

## DISCUSSION

### Main Findings

This study examined depressive symptoms and physical activity as potential mediators of the longitudinal relationship between baseline usual presence of pain or discomfort and functional dependence over a mean follow-up of 6.3 years in people with arthritis. Our results provide empirical evidence for a total effect of baseline pain on future functional dependence in ADLs and IADLs. Notably, 23.3% of this total effect was mediated by depressive symptoms and physical activity. However, as discussed earlier, estimates of direct and indirect effects may not sum exactly to the total effect, and this percentage should be interpreted as approximate. Consistent with the biopsychosocial approach to pain in rehabilitation,^38^ these findings highlight the importance of addressing both psychological and behavioral pathways in rehabilitation programs for patients with arthritis.

### Comparison With the Literature

Our results indicate that individuals experiencing pain at baseline had higher odds of functional dependence at follow-up, while adjusting for sex, age, and baseline functional dependence. This direct association between baseline pain and future functional dependence aligns with previous research demonstrating that pain is a key determinant of disability in individuals with arthritis.^3–6^ Taken together, these results suggests that early pain management may be critical in preventing long-term declines in functional abilities of individuals with arthritis.

Depressive symptoms were found to mediate the relationship between pain and functional dependence, accounting for 69.5% of the indirect effect. Specifically, our results suggest that individuals who reported being usually in pain or discomfort at baseline were more likely to experience depressive symptoms, which in turn increased the odds of functional dependence at follow-up. These results are consistent with those from studies of low back pain and post-traumatic pain that have documented the mediating role of depressive symptoms in the relationship between pain and functional dependence.^28–35^ Importantly, our findings extend this evidence to individuals with arthritis, highlighting the broader relevance of these mechanisms across pain-related conditions. As depression is both modifiable and treatable, these findings suggest that rehabilitation professionals should consider mental health when implementing interventions aimed at pain-related functional decline in individuals with arthritis.

Physical activity was also a mediator, accounting for 27.0% of the total indirect effect of pain on functional dependence. This finding is consistent with studies showing similar indirect effects in individuals with low back pain.^46^^,^^47^ Importantly, our findings extend this evidence to individuals with arthritis, reinforcing the importance of physical activity in maintaining functional independence in this population. The mediating effect is consistent with recent work in osteoarthritis showing that individuals with stronger tendencies to approach than avoid physical activity are less likely to reduce activity levels, even when experiencing high fear of movement.^65^ These results suggest that targeting both fear and motivational attitudes may improve physical activity and functional outcomes. Accordingly, exercise therapy should be considered as a component of rehabilitation programs aimed at restoring or preserving ADL and IADL independence in individuals with arthritis.

The mediation model we specified assumed a serial pathway in which depressive symptoms contribute to lower physical activity, which in turn affects functional dependence. The analysis yielded statistical evidence consistent with this hypothesized pathway. Although this serial effect accounted for only 3.5% of the total indirect effect, it suggests that individuals who develop depressive symptoms due to pain may subsequently reduce their physical activity, further exacerbating their risk of functional decline.

Our sensitivity analyses confirmed the robustness of our findings across different functional dependence classifications, statistical approaches, and arthritis subtypes. Particularly, analyses restricted to participants with osteoarthritis showed comparable mediation patterns, suggesting that the observed effects apply to the most common form of arthritis. However, in rheumatoid arthritis, the mediation effect was primarily driven by depressive symptoms, while the indirect effects through physical activity and the serial pathway were not statistically significant. This lack of significance may be due to the lower statistical power of this subsample but may also suggest that the mechanisms linking pain to functional decline differ between arthritis subtypes and warrant further investigation.

### Strengths and Limitations

This study has several strengths, including a large sample size of 6972 participants, a long follow-up period ranging from 4.0 to 9.6 years after baseline, the use of multiple mediation analyses, and bootstrapping to robustly estimate the significance of indirect effects. Several limitations should also be noted. First, the outcome and mediator variables were self-reported, which may have introduced measurement bias, especially in the context of a chronic condition like arthritis with a gradual onset. However, this limitation is common and often unavoidable in large cohort studies. Second, despite adjusting for key confounders, unmeasured variables such as medication use, disease severity, and comorbidities could have influenced the results. Third, since depressive symptoms and physical activity were both assessed at baseline, our mediation estimates may reflect concurrent associations rather than true causal pathways. Fourth, while CLSA has implemented sampling strategy designed to produce a nationally representative sample of adults aged 45 to 85 years at the time of recruitment, selection bias cannot be ruled out and may limit the generalizability of findings to individuals without access to a telephone, the internet, proficiency in English or French, or the ability to travel to a data collection site. Fifth, the operationalization of pain was based on a single binary item asking whether participants were usually free of pain or discomfort. This phrasing does not directly measure the presence, intensity, or duration of pain and may be subject to interpretation. Finally, although mediation analysis provides insight into potential causal pathways, the observational nature of the study precludes definitive causal conclusions.

## CONCLUSION

This study provides evidence suggesting that experiencing ongoing pain or discomfort contributes to long-term functional dependence both directly and indirectly through depressive symptoms and physical inactivity. The direct effect shows that individuals reporting pain have higher odds of being functionally dependent in ADLs and IADLs compared to those without pain. In addition, depressive symptoms and physical inactivity mediate this relationship.

Our findings underscore the need for a comprehensive approach to arthritis care that goes beyond pain management alone. They suggest that interventions should include strategies to address depressive symptoms and promote physical activity to mitigate the long-term impact of pain on functional dependence. For physical therapist practice, this means integrating routine screening for depressive symptoms into assessments and incorporating psychological support or referral pathways where needed. Furthermore, physical therapists should tailor exercise interventions to not only accommodate pain but also to enhance adherence and motivation, particularly in individuals exhibiting depressive symptoms, who may be at risk of physical inactivity.^66^ To support sustained engagement in physical activity, it is important to recognize that promoting health benefits alone is often insufficient.^67^ Instead, interventions should aim to foster pleasure and positive affective experiences related to physical activity.^68^

A biopsychosocial framework that integrates physical, psychological, and behavioral components may provide a more effective approach to maintaining, restoring, or improving physical function in individuals with arthritis. Clinically, this may involve interdisciplinary collaboration with mental health professionals and the use of behavior change techniques, such as goal setting, motivational interviewing, and graded activity, to promote physical activity despite pain.

## Supplementary Material

2025-0137_R2_Unmasked_Supplementary_CLSA_arthritis_pzaf120

## Data Availability

In accordance with good research practices,^69^ the R scripts used to analyse the data are publicly available in Zenodo.^58^ This research was made possible using the data collected by the Canadian Longitudinal Study on Aging (CLSA) and was conducted using the CLSA Baseline Comprehensive Dataset version 7.0, Follow-Up 1 Comprehensive Dataset version 5.0, and Follow-Up 2 Comprehensive dataset version 2.0 under application number 2304015. This dataset is available for researchers who meet the criteria (www.clsa-elcv.ca). This manuscript was posted before peer review on the MedRxiv preprint repository on February 12, 2025.^70^

## REPORTING GUIDELINES

This manuscript conforms to the AGReMA guidelines for mediation analyses of observational studies.^71^

## References

[1] Qiao L, Li M, Deng F, et al. Epidemiological trends of osteoarthritis at the global, regional, and national levels from 1990 to 2021 and projections to 2050. medRxiv. 2024;Version 2:1–37. 10.1101/2024.06.30.24309697 PMC12570834 41152934

[2] GBD 2021 osteoarthritis collaborators. Global, regional, and national burden of osteoarthritis, 1990-2020 and projections to 2050: a systematic analysis for the global burden of disease study 2021. Lancet Rheumatol. 2023;5(9):e508–e522. 10.1016/S2665-9913(23)00163-7 37675071 PMC10477960

[3] Sharma L, Cahue S, Song J, Hayes K, Pai YC, Dunlop D. Physical functioning over three years in knee osteoarthritis: role of psychosocial, local mechanical, and neuromuscular factors. Arthritis Rheum. 2003;48(12):3359–3370. 10.1002/art.11420 14673987

[4] van Dijk GM, Veenhof C, Spreeuwenberg P, et al. Prognosis of limitations in activities in osteoarthritis of the hip or knee: a 3-year cohort study. Arch Phys Med Rehabil. 2010;91(1):58–66. 10.1016/j.apmr.2009.08.147 20103397

[5] Pisters MF, Veenhof C, van Dijk GM, Heymans MW, Twisk JW, Dekker J. The course of limitations in activities over 5 years in patients with knee and hip osteoarthritis with moderate functional limitations: risk factors for future functional decline. Osteoarthr Cartil. 2012;20(6):503–510. 10.1016/j.joca.2012.02.002 22330176

[6] van Allen ZM, Boisgontier MP. Prospective classification of functional dependence: insights from machine learning and the Canadian longitudinal study on aging. Physiother Can. 2025. 10.3138/ptc-2024-0136

[7] Vlaeyen JWS, Linton SJ. Fear-avoidance model of chronic musculoskeletal pain: 12 years on. Pain. 2012;153(6):1144–1147. 10.1016/j.pain.2011.12.009 22321917

[8] Holla JF, Sanchez-Ramirez DC, van der Leeden M, et al. The avoidance model in knee and hip osteoarthritis: a systematic review of the evidence. J Behav Med. 2014;37(6):1226–1241. 10.1007/s10865-014-9571-8 24841177

[9] Goubran M, Farajzadeh A, Lahart IM, Bilodeau M, Boisgontier MP. Relationship between fear of movement and physical activity in patients with cardiac, rheumatologic, neurologic, pulmonary, or pain conditions: a systematic review and meta-analysis. Phys Ther. 2025;105(6):pzaf050. 10.1093/ptj/pzaf050 40188486 PMC12207065

[10] Araujo IL, Castro MC, Daltro C, Matos MA. Quality of life and functional independence in patients with osteoarthritis of the knee. Knee Surg Relat Res. 2016;28(3):219–224. 10.5792/ksrr.2016.28.3.219 27595076 PMC5009047

[11] Sieber S, Roquet A, Lampraki C, Jopp DS. Multimorbidity and quality of life: the mediating role of ADL, IADL, loneliness, and depressive symptoms. Innov Aging. 2023;7(4):1–13. 10.1093/geroni/igad047 PMC10332504 37435089

[12] Falck RS, Percival AG, Tai D, Davis JC. International depiction of the cost of functional independence limitations among older adults living in the community: a systematic review and cost-of-impairment study. BMC Geriatr. 2022;22(1):815. 10.1186/s12877-022-03466-w 36273139 PMC9587635

[13] Angst F, Benz T, Lehmann S, et al. Extended overview of the longitudinal pain-depression association: a comparison of six cohorts treated for specific chronic pain conditions. J Affect Disord. 2020;273:508–516. 10.1016/j.jad.2020.05.044 32560947

[14] Wilcox S, Der Ananian C, Abbott J, et al. Perceived exercise barriers, enablers, and benefits among exercising and nonexercising adults with arthritis: results from a qualitative study. Arthritis Rheum. 2006;55(4):616–627. 10.1002/art.22098 16874785

[15] Petursdottir U, Arnadottir SA, Halldorsdottir S. Facilitators and barriers to exercising among people with osteoarthritis: a phenomenological study. Phys Ther. 2010;90(7):1014–1025. 10.2522/ptj.20090217 20466741

[16] Gay C, Eschalier B, Levyckyj C, Bonnin A, Coudeyre E. Motivators for and barriers to physical activity in people with knee osteoarthritis: a qualitative study. Joint Bone Spine. 2018;85(4):481–486. 10.1016/j.jbspin.2017.07.007 28757341

[17] Boisgontier MP, Orsholits D, von Arx M, et al. Adverse childhood experiences, depressive symptoms, functional dependence, and physical activity: a moderated mediation model. J Phys Act Health. 2020;17(8):790–799. 10.1123/jpah.2019-0133 32698122

[18] Penninx BW, Messier SP, Rejeski WJ, et al. Physical exercise and the prevention of disability in activities of daily living in older persons with osteoarthritis. Arch Intern Med. 2001;161(19):2309–2316. 10.1001/archinte.161.19.2309 11606146

[19] Merry Del Val B, Shukla SR, Oduoye MO, Nsengiyumva M, Tesfaye T, Glinkowski WM. Prevalence of mental health disorders in knee osteoarthritis patients: a systematic review and meta-analysis. Ann Med Surg. 2024;86(8):4705–4713. 10.1097/MS9.0000000000002258 PMC11305717 39118762

[20] Hill J, Harrison J, Christian D, et al. The prevalence of comorbidity in rheumatoid arthritis: a systematic review and meta-analysis. Br J Community Nurs. 2022;27(5):232–241. 10.12968/bjcn.2022.27.5.232 35522453

[21] Fonseca-Rodrigues D, Rodrigues A, Martins T, et al. Correlation between pain severity and levels of anxiety and depression in osteoarthritis patients: a systematic review and meta-analysis. Rheumatology. 2021;61(1):53–75. 10.1093/rheumatology/keab512 34152386

[22] Kroenke K, Wu J, Bair MJ, Krebs EE, Damush TM, Tu W. Reciprocal relationship between pain and depression: a 12-month longitudinal analysis in primary care. J Pain. 2011;12(9):964–973. 10.1016/j.jpain.2011.03.003 21680251 PMC3222454

[23] Sayre EC, Esdaile JM, Kopec JA, et al. Specific manifestations of knee osteoarthritis predict depression and anxiety years in the future: Vancouver longitudinal study of early knee osteoarthritis. BMC Musculoskelet Disord. 2020;21(1):467. 10.1186/s12891-020-03496-8 32677938 PMC7367326

[24] Zheng S, Tu L, Cicuttini F, et al. Depression in patients with knee osteoarthritis: risk factors and associations with joint symptoms. BMC Musculoskelet Disord. 2021;22(1):40. 10.1186/s12891-020-03875-1 33413273 PMC7791830

[25] Li M, Nie Y, Zeng Y, et al. The trajectories of depression symptoms and comorbidity in knee osteoarthritis subjects. Clin Rheumatol. 2022;41(1):235–243. 10.1007/s10067-021-05847-9 34490511

[26] Rathbun AM, Stuart EA, Shardell M, Yau MS, Baumgarten M, Hochberg MC. Dynamic effects of depressive symptoms on osteoarthritis knee pain. Arthritis Care Res. 2018;70(1):80–88. 10.1002/acr.23239 PMC5607075 28320048

[27] Jacobs CA, Vranceanu AM, Thompson KL, Lattermann C. Rapid progression of knee pain and osteoarthritis biomarkers greatest for patients with combined obesity and depression: data from the osteoarthritis initiative. Cartilage. 2020;11(1):38–46. 10.1177/1947603518777577 29855190 PMC6921961

[28] Hall AM, Kamper SJ, Maher CG, Latimer J, Ferreira ML, Nicholas MK. Symptoms of depression and stress mediate the effect of pain on disability. Pain. 2011;152(5):1044–1051. 10.1016/j.pain.2011.01.014 21306826

[29] Seekatz B, Meng K, Faller H. Depressivity as mediator in the fear-avoidance model: a path analysis investigation of patients with chronic back pain [German]. Schmerz. 2013;27(6):612–618. 10.1007/s00482-013-1376-0 24337428

[30] Marshall PWM, Schabrun S, Knox MF. Physical activity and the mediating effect of fear, depression, anxiety, and catastrophizing on pain related disability in people with chronic low back pain. PLoS One. 2017;12(7):e0180788. 10.1371/journal.pone.0180788 28686644 PMC5501599

[31] Garcia AN, Cook CE, Gottfried O. Psychological, mobility, and satisfaction variables mediate the relationship between baseline back pain intensity and long-term outcomes in individuals who underwent lumbar spine surgery. Musculoskelet Sci Pract. 2021;55:102424. 10.1016/j.msksp.2021.102424 34246843

[32] Wegener ST, Castillo RC, Haythornthwaite J, EJ MK, Bosse MJ, LEAP Study Group. Psychological distress mediates the effect of pain on function. Pain. 2011;152(6):1349–1357. 10.1016/j.pain.2011.02.020 21396777

[33] Ross C, Juraskova I, Lee H, et al. Psychological distress mediates the relationship between pain and disability in hand or wrist fractures. J Pain. 2015;16(9):836–843. 10.1016/j.jpain.2015.05.007 26072389

[34] Talaei-Khoei M, Fischerauer SF, Jha R, Ring D, Chen N, Vranceanu AM. Bidirectional mediation of depression and pain intensity on their associations with upper extremity physical function. J Behav Med. 2018;41(3):309–317. 10.1007/s10865-017-9891-6 28986704

[35] Probst T, Neumeier S, Altmeppen J, Angerer M, Loew T, Pieh C. Depressed mood differentially mediates the relationship between pain intensity and pain disability depending on pain duration: a moderated mediation analysis in chronic pain patients. Pain Res Manag. 2016;2016:3204914. 10.1155/2016/3204914 27445605 PMC4904642

[36] Hawker GA, Gignac MA, Badley E, et al. A longitudinal study to explain the pain-depression link in older adults with osteoarthritis. Arthritis Care Res. 2011;63(10):1382–1390. 10.1002/acr.20298 20662042

[37] Wang Q, Jayasuriya R, Man WY, Fu H. Does functional disability mediate the pain-depression relationship in older adults with osteoarthritis? A longitudinal study in China. Asia Pac J Public Health. 2015;27(2):NP382–NP391. 10.1177/1010539512443974 22535552

[38] Smart KM . The biopsychosocial model of pain in physiotherapy: past, present and future. Phys Ther Rev. 2023;28(2):61–70. 10.1080/10833196.2023.2177792

[39] Treadway MT, Zald DH. Reconsidering anhedonia in depression: lessons from translational neuroscience. Neurosci Biobehav Rev. 2011;35(3):537–555. 10.1016/j.neubiorev.2010.06.006 20603146 PMC3005986

[40] Katz P . Fatigue in rheumatoid arthritis. Curr Rheumatol Rep. 2017;19(5):25. 10.1007/s11926-017-0649-5 28386762

[41] Fawole HO, Riskowski JL, Dell'Isola A, et al. Determinants of generalized fatigue in individuals with symptomatic knee osteoarthritis: the MOST study. Int J Rheum Dis. 2020;23(4):559–568. 10.1111/1756-185X.13797 31991526 PMC7160026

[42] Cijs B, Stekelenburg R, Veenhof C, et al. Prognostic factors and changes in pain, physical functioning, and participation in patients with hip and/or knee osteoarthritis: a systematic review. Arthritis Care Res. 2025;77(2):228–239. 10.1002/acr.25428 PMC11771565 39228051

[43] Torlinska B, Raza K, Filer A, et al. Predictors of quality of life, functional status, depression and fatigue in early arthritis: comparison between clinically suspect arthralgia, unclassified arthritis and rheumatoid arthritis. BMC Musculoskelet Disord. 2024;25(1):307. 10.1186/s12891-024-07446-6 38643104 PMC11031996

[44] Burrows NJ, Barry BK, Sturnieks DL, Booth J, Jones MD. The relationship between daily physical activity and pain in individuals with knee osteoarthritis. Pain Med. 2020;21(10):2481–2495. 10.1093/pm/pnaa096 32330278

[45] Fransen M, McConnell S, Harmer AR, Van der Esch M, Simic M, Bennell KL. Exercise for osteoarthritis of the knee: a Cochrane systematic review. Br J Sports Med. 2015;49(24):1554–1557. 10.1136/bjsports-2015-095424 26405113

[46] Ekediegwu EC, OK OCVO. Pain intensity, physical activity, quality of life, and disability in patients with mechanical low back pain: a cross-sectional study. Bull Fac Phys Ther. 2024;29:1(2024):1–9. 10.1186/s43161-023-00167-2

[47] Karklins AE, Pernaa KI, Saltychev M, Juhola JE, Arokoski JPA. Physical activity as mediator between back pain and disability. Int J Rehabil Res. 2024;47(3):192–198. 10.1097/MRR.0000000000000638 39036996

[48] Goh SL, Persson MSM, Stocks J, et al. Efficacy and potential determinants of exercise therapy in knee and hip osteoarthritis: a systematic review and meta-analysis. Ann Phys Rehabil Med. 2019;62(5):356–365. 10.1016/j.rehab.2019.04.006 31121333 PMC6880792

[49] Raina PS, Wolfson C, Kirkland SA. Canadian longitudinal study on aging (CLSA). *Protocol*. 2009:1–128. Available from: https://www.clsa-elcv.ca/wp-content/uploads/2023/06/combinedprotocol_v3_2013_for_web.pdf 10.1017/S0714980809990055 19860977

[50] Wolfson C, Raina PS, Kirkland SA, et al. The Canadian community health survey as a potential recruitment vehicle for the Canadian longitudinal study on aging. Can J Aging. 2009;28(3):243–249. 10.1017/S0714980809990031 19860979

[51] Fillenbaum GG . Multidimensional Functional Assessment of Older Adults: The Duke Older Americans Resources and Services Procedures. 1st ed. New York, NY: Psychology Press; 1988, 10.4324/9780203771563

[52] Fillenbaum GG, Smyer MA. The development, validity, and reliability of the OARS multidimensional functional assessment questionnaire. J Gerontol. 1981;36(4):428–434. 10.1093/geronj/36.4.428 7252074

[53] Statistics Canada . *Canadian community health survey, 2007-2008*. Abacus data. Network. 2009;Version 1:1–96. Available from: https://hdl.handle.net/11272.1/AB2/UITY8E

[54] Andresen EM, Malmgren JA, Carter WB, Patrick DL. Screening for depression in well older adults: evaluation of. Am J Prev Med. 1994;10(2):77–84. 10.1016/S0749-3797(18)30622-6 8037935

[55] Washburn RA, Smith KW, Jette AM, Janney CA. The physical activity scale for the elderly (PASE): development and evaluation. J Clin Epidemiol. 1993;46(2):153–162. 10.1016/0895-4356(93)90053-4 8437031

[56] New England Research Institutes . PASE: Physical Activity Scale for the Elderly: administration and Scoring Instruction Manual. 1991:1–24. Available from: https://meetinstrumentenzorg.nl/wp-content/uploads/instrumenten/PASE-handl.pdf

[57] R Core Team . R: A Language and Environment for Statistical Computing. Vienna, Austria: R Foundation for Statistical Computing; 2024.

[58] van Allen ZM, Boisgontier MP. Depressive symptoms and physical activity mediate the effect of pain on ADL and IADL functional independence in adults with arthritis: R script. Zenodo. 2025;Version 1.1. 10.5281/zenodo.14847424

[59] Hayes AF . Introduction to Mediation, Moderation, and Conditional Process Analysis: a Regression-Based Approach. 3rd ed. New York, NY: The Guilford Press; 2022.

[60] Yzerbyt V, Muller D, Batailler C, Judd CM. New recommendations for testing indirect effects in mediational models: the need to report and test component paths. J Pers Soc Psychol. 2018;115(6):929–943. 10.1037/pspa0000132 30550319

[61] Iacobucci D . Mediation analysis and categorical variables: the final frontier. J Consum Psychol. 2012;22(4):582–594. 10.1016/j.jcps.2012.03.006 PMC3501728 23180961

[62] Xu S, Coffman DL, Luta G, Niaura RS. Tutorial on causal mediation analysis with binary variables: an application to health psychology research. Health Psychol. 2023;42(11):778–787. 10.1037/hea0001299 37410423 PMC10615709

[63] Tofighi D, Kelley K. Indirect effects in sequential mediation models: evaluating methods for hypothesis testing and confidence interval formation. Multivariate Behav Res. 2020;55(2):188–210. 10.1080/00273171.2019.1618545 31179751 PMC6901816

[64] Rosseel Y, Jorgensen TD, De Wilde L. lavaan: latent variable analysis. Version 0.6-19. Published September 2024. Accessed May 2025. https://cran.r-project.org/package=lavaan

[65] Goubran M, Zammar C, Tellez Alvarez S, et al. Approach-avoidance tendencies influence the relationship between fear of movement and physical activity in osteoarthritis. *Physiotherapy Canada*. 2025. 10.3138/ptc-2025-0011

[66] Boisgontier MP, Iversen MD. Physical Inactivity: A Behavioral Disorder in the Physical Therapist's Scope of Practice. Phys Ther. 2020;100(5):743–746. 10.1093/ptj/pzaa011 31944246

[67] Maltagliati S, Sarrazin P, Fessler L, Lebreton M, Cheval B. Why people should run after positive affective experiences instead of health benefits. J Sport Health Sci. 2024;13(4):445–450. 10.1016/j.jshs.2022.10.005 36334885 PMC11184383

[68] Ekkekakis P . People have feelings! Exercise psychology in paradigmatic transition. Curr Opin Psychol. 2017;16:84–88. 10.1016/j.copsyc.2017.03.018 28813362

[69] Boisgontier MP . Research integrity requires to be aware of good and questionable research practices. Eur Rehabil J. 2021;2(1):1–3. 10.52057/erj.v2i1.24

[70] Goubran M, van Allen ZM, Bilodeau M, Boisgontier MP. Depressive symptoms and physical activity mediate the adverse effect of pain on functional independence in patients with arthritis: evidence from the Canadian longitudinal study on aging. MedRxiv. 2025;Version 2:1–23. 10.1101/2025.02.10.25322043 PMC12613829 41042239

[71] Lee H, Cashin AG, Lamb SE, et al. A guideline for reporting mediation analyses of randomized trials and observational studies: the AGReMA statement. JAMA. 2021;326(11):1045–1056. 10.1001/jama.2021.14075 34546296 PMC8974292

