# Supplementary material for: Depressive Symptoms and Physical Activity Mediate the Adverse Effect of Pain on Functional Dependence in Patients With Arthritis: Evidence From the Canadian Longitudinal Study on Aging
Source: Phys Ther. 2025 Oct 3;105(11):pzaf120. doi: 10.1093/ptj/pzaf120 (PMC12613829; doi:10.1093/ptj/pzaf120)
Supplement: 2025-0137_R2_Unmasked_Supplementary_CLSA_arthritis_pzaf120 [file 2025-0137_r2_unmasked_supplementary_clsa_arthritis_pzaf120.pdf]

SUPPLEMENTARY MATERIAL

Depressive symptoms and physical activity mediate the adverse effect of pain on functional dependence in patients with arthritis: Evidence from the Canadian Longitudinal Study on Aging

Miriam Goubran, PhDc[1,2,†], Zachary M. van Allen, MBA, PhD[1,3,†], Martin Bilodeau, PT, PhD[1,2], Matthieu P. Boisgontier, PT, PhD, HDR[1,2,3,4,*]

[1]Faculty of Health Sciences, University of Ottawa, Canada; [2]Bruyère Health Research Institute, Ottawa, Canada; [3]Perley Health Centre of Excellence in Frailty-Informed Care, Ottawa, Canada; [4]Institut du Savoir Montfort, Hôpital Montfort, Ottawa, Canada. [†]MG and ZMvA contributed equally to this work. [*]Corresponding author: matthieu.boisgontier@uOttawa.ca

Suppl. Material 1. Scoring scheme for the Physical Activity Scale for the Elderly (PASE)

Suppl. Material 2. Sensitivity analysis based on structural equation modeling (SEM)

Supplementary Material 1. Scoring scheme for the Physical Activity Scale for the Elderly (PASE).

PASE Item	Type of Activity	Activity Weight	Activity Frequency	Weight times Frequency
2.	Walk outside home	20	a.	
3.	Light sport / recreational activities	21	a.	
4.	Moderate sport / recreational activities	23	a.	
5.	Strenuous sport / recreational activities	23	a.	
6.	Muscle strength / endurance exercises	30	a.	
7.	Light housework	25	b.	
8.	Heavy housework or chores	25	b.	
9a.	Home repairs	30	b.	
9b.	Lawn work or yard care	36	b.	
9c.	Outdoor gardening	20	b.	
9d.	Caring for another person	35	b.	
10.	Work for pay or as volunteer	21	c.	
			PASE SCORE:	

Activity Frequency Values:

a. Use hours per day conversion table below
b. 1 = activity reported in past week, 0 = activity not reported
c. Divide work hours reported in Item 10.1 by seven; if no work hours or if job involves mainly sitting with slight arm movements (Item 10.2 = 1), then activity frequency = 0.

ACTIVITY TIME TO HOURS PER DAY CONVERSION TABLE

Days of Activity	Hours Per Day of Activity	Hours Per Day
0. Never		0
1. Seldom	1. Less than 1 hour	.11
	2. 1-2 hours	.32
	3. 2-4 hours	.64
	4. More than 4 hours	1.07
2. Sometimes	1. Less than 1 hour	.25
	2. 1-2 hours	.75
	3. 2-4 hours	1.50
	4. More than 4 hours	2.50
3. Often	1. Less than 1 hour	.43
	2. 1-2 hours	1.29
	3. 2-4 hours	2.57
	4. More than 4 hours	4.29

Full administration and scoring instruction manual available at https://meetinstrumentenzorg.nl/wp-content/uploads/instrumenten/PASE-handl.pdf

Supplementary Material 2. Sensitivity analysis based on structural equation modeling (SEM)

As a sensitivity analysis, we conducted a serial mediation model using structural equation modeling (SEM) with the *lavaan* package in R. The model specifies paths from pain to depressive symptoms and physical activity, with both mediators modeled as predictors of functional dependence at follow-up. Covariates included sex, age, and baseline functional dependence. The outcome was treated as an ordered categorical variable, and the model was estimated using the Weighted Least Squares Mean and Variance (WLSMV) estimator, which is appropriate for binary outcomes. Indirect effects were computed for three pathways: through depressive symptoms, through physical activity, and through the serial path.

Results showed that the direct effect of baseline pain on functional dependence at follow-up (c') remained statistically significant after accounting for both mediators (log OR = 0.270, $p < .001$). In addition, the indirect effect of pain on functional dependence was statistically significant (log OR = 0.077, $p < .001$). This indirect effect was composed of three distinct pathways. The indirect effect through depressive symptoms alone was statistically significant (log OR = 0.056, $p < .001$), as was the indirect effect through physical activity alone (log OR = 0.018, $p = .001$). The serial pathway also yielded a small but significant indirect effect (log OR = 0.002, $p = .008$). The total effect of pain on functional dependence, combining both direct and indirect components, was estimated at log OR = 0.347 ($p < .001$). The model explained 4.7% of the variance in depressive symptoms, 25.4% of the variance in physical activity, and 21.3% of the variance in functional dependence.

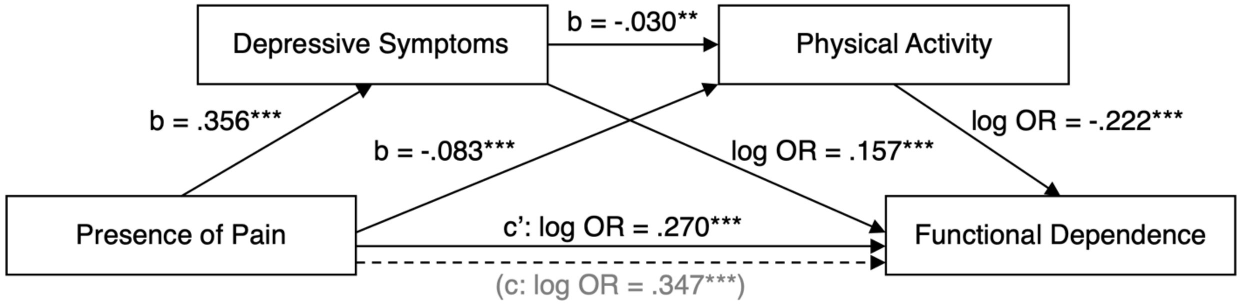

Suppl. Figure 1. Serial multiple mediation model based on structural equation modeling using the *lavaan* package.
